# EU After COVID-19: An Opportunity for Policy Coordination

**DOI:** 10.1007/s10272-021-0982-9

**Published:** 2021-08-05

**Authors:** Antonia Díaz, Luis A. Puch

**Affiliations:** 1 Department of Ecomomy, Universidad Carlos III de Madrid, C/Madrid 126, 28903 Getafe (Madrid), Spain; 2 Department of Economic Analysis and Quantitative Economics, Universidad Complutense de Madrid, Campus de Somosaguas, 28223 Pozuelo de Alarcón (Madrid), Spain

## Abstract

Despite all the difficulties inherent to our political organisation, the European Union has taken a bold step by doubling the EU budget for the next six years with the NGEU fund.

In order to mitigate the impact of the COVID-19 pandemic, most G20 member countries have announced fiscal stimulus of significant magnitude. Particularly, the United States has passed two packages and authorised additional aid that amounts to approximately $5 trillion. The sheer magnitude of the package raises concerns about the effectiveness of the European measures; in particular, the unprecedented EU-wide fiscal stimulus plan included in the Next Generation EU (NGEU) fund. In this contribution, we argue that traditional fiscal stimulus, which works through demand channels, is not what the European Union needs. We need to strengthen our common fiscal capacity and improve policy coordination; in particular, sectoral policies that support the industry and flow downstream to the services sector. This policy is very much in line with the course of action taken by the US and the leading Asian countries to increase potential growth.

## Political institutions and economic policy outcomes

Any study of the effects of EU-wide policy should take into account the fact that the European Union is not a country but a confederation in progress, which, depending on the views of the observer, can be thought of as going too quickly or too slowly. If we look at the EU in this way, through the federation lenses, we see that ours has an uneasy balance between the federal government, i.e. the European Commission, and the representation of the states, i.e. the European Council. This is particularly clear when we compare the EU with the United States, where the division of state and federal powers is clear or, at least, has been tested by 200 years of litigation, debates and political interaction. Moreover, the size of the European budget is tiny, just 1% of the EU's GDP, compared to the revenues of the US federal government, which were about 16.3% of their GDP in 2018. This comparison is not informative because it is due mainly to the fact that the US federal government collects all taxes and Social Security revenues, whereas the EU budget is comprised, mostly, of country contributions. On top of that, the US federal government controls those policy tools that enhance risk sharing across the federation. We are referring not only to Social Security, Medicaid and Medicare but to the Welfare State and all the tools of sectoral policy. We are referring to banking and financial markets supervision, too. The federal government of the US controls the policies and the fiscal resources needed to finance them. But both sides of policy should go together: "expenditure" capacity and "fiscal" capacity. This is so because the former without the latter creates moral hazard problems, as it gives incentives to profligacy in member states (Díaz, 2020, 2021).

This context is necessary to study and compare US and EU policy responses to the COVID-19 pandemic. The fiscal measures taken by the US government during 2020 amount to more than 14% of GDP, which are going to be topped by a new package in 2021, the size of which is committed to be about another 11% of GDP. The EU countries' responses are comparable in size but very heterogeneous. For instance, according to Anderson et al. (2020) and the International Monetary Fund (2021), the direct fiscal measures in Germany in 2020 amount to 15.6% of its GDP, along with guarantees worth 24.3% of GDP; Spain, on the other hand, has committed 8.1% and 13.3% of its GDP in direct fiscal measures and guarantees, respectively. The EU grants, especially those coming from the Recovery and Resilience Facility, may amount to 1.8% of Spanish GDP annually for the period 2021-27. Therefore, the size of all responses is similar. Nevertheless, there are differences in composition and the institutions in charge of conducting those policies. Those differences make it difficult to compare their effects.

First, European governments have set extensive furlough schemes and guarantees so that workers remain attached to their employers and firms do not declare bankruptcy, whereas the US has allowed jobs and firms to be destroyed. We think that those differences are consistent with the fact that firm creation is more troublesome in Europe and firms depend more on bank financing.

**Antonia Díaz**, Universidad Carlos III de Madrid, Spain.

**Luis A. Puch**, Universidad Complutense de Madrid, Spain.

Thus, it is a policy whose benefit can only be measured by knowing the counterfactual. Conversely, the US has focused on family and emergency assistance, as its automatic stabilisers are smaller than in Europe. Moreover, although we are not in a position to estimate its effect, the fact that health systems and coverage vary so much suggests that medium-run effects on health and labour productivity may be sizeable, particularly among low-skilled workers.

Second, in line with the federation analogy, the US federal government controls the policy measures to mitigate the effect of COVID-19, whereas in the EU most of the funds are controlled by the states. This distinction makes the EU policy, most likely, inefficient. This is so because uncoordinated fiscal aid to firms implies that different countries may use different criteria for aiding firms without internalising the effect on competitors established in other countries. That is, as Motta and Peitz (2020) argue, uncoordinated aid policies distort the European level playing field for firms and harm the Single Market. The negative effects of uncoordinated policy do not come only from the supply side. They also come from the demand side, as most of the trade of EU members takes place inside the Union.

This is particularly unfortunate, as the sectoral composition of the EU makes it more vulnerable to the COVID-19 pandemic than the US. For instance, the share of the tour- ism sector in aggregate value added and employment is two percentage points higher than the OECD average, while the US is below the OECD average (OECD, 2020). Moreover, the share of manufacturing in GDP is almost three percentage points higher in the EU than in the US, which implies that Europe is more affected by global supply chain disruptions than the US. The evidence suggests that, indeed, this has been the case. According to the Bureau of Economic Analysis, the US GDP fell in the fourth quarter of 2020 by 2.39% with respect to the same quarter of the previous year, whereas EU GDP shrank by 4.44% (on quarterly data basis). Not only that; the US has already grown during the first quarter of 2021, 0.4%, whereas EU GDP had an interannual growth rate of -1.2% (again, using quarterly data). This difference in the first quarter of 2021 is most likely due to the hesitant starting pace of vaccination in Europe compared to the US, and delivery delays due to not thoroughly specified contracts with vaccine suppliers. This might very well be the aggregate cost of having a common institution that is inexperienced in coordinating policies. Learning by doing should change this situation.

The importance of strengthening the institutions in charge of coordination policy appears to be more urgent when we take into account two current developments. The first one is due to the asymmetric sectoral impact of COVID-19. The second one is related to the rapid technological change that we are experiencing. Digital sectors are the absolute winners of the COVID-19 crisis. Consumption patterns have shifted from consuming services locally to consuming them through digital platforms, which are, for the most part, US-based. This must have some impact on the tax base of EU countries. In addition, this crisis has burst in a moment of rapid technical change: Think of electric cars and 5G technology, where the US and China are the leaders. Participating in this international innovation race requires a coordinated effort by all EU members.

## Fiscal stimulus for resilience and recovery in a quasi-federation

Despite all the difficulties inherent to our political organisation, the European Union has taken a bold step by doubling the EU budget for the next six years with the NGEU fund. To access the resources of the fund, EU members present their recovery plans to the Commission with specific details about policies, milestones, deadlines and possible outcomes. That is, EU members must present quantitative forecasts of the effects of the policies funded by NGEU. In the meantime, President Biden has signed a stimulus package, the sheer size of which has ignited a lively discussion on the likely effect of fiscal impulses on both sides of the Atlantic.

The question about whether government expenditure raises aggregate output has been largely studied in the so-called empirical fiscal multipliers literature. The most popular view of the fiscal multiplier is the textbook example in which the real interest rate is constant. However, constant real interest rates depend very much on how monetary policy has been conducted in the past, as Cloyne et al. (2020) show. Moreover, effects of fiscal multipliers may be lower in a currency union (the majority of the European Union's member states) than in a single country, like the US. There are two reasons for that. First, a real reason: As Farih and Werning (2016) show, fiscal stimuli may change the regional distribution of the demand and, therefore, real exchange rates, which affects competitiveness and, in turn, may offset the intended effect of the policy. The second reason is financial. As Bianchi et al. (2019) point out, it is not clear at all that real interest rates may remain constant in all countries in the EMU due to perceived changes in sovereign risk. It will be likely that the ECB would have to pursue an even more active policy to counteract such risks.

There is another reason why demand policies (such as traditional fiscal stimuli) may not be as effective in the European Union as in a single country. According to estimates (see Auerbach et al., 2019; or Dupor and Guerrero, 2017), regional fiscal multipliers are lower than the national multiplier in the US. Canova and Pappa (2021) find the opposite for the EU when estimating the effect of EU regional structural funds. This evidence suggests that spillovers work in opposite directions because of a lack of policy coordination of EU members. This points to the fact that the European Union should concentrate on supply-side policies as a very basic supply-side policy is strengthening our common institutions and policy coordination.

The current discussion on the effects of fiscal stimuli has also been prompted by the fears of inflation. It is true that we are already seeing inflation rising because of COVID-19 related global supply chain disruptions. Temporary shortages may trigger more difficult issues of strategic behaviour coming from imperfect competition. Economy ministers in EU countries and the US are betting on this rise of inflation being transitory, and its duration will depend much on geopolitics, as we are seeing in the case of semiconductor shortages (see Paduano, 2021). There is also the fear that the fiscal stimuli themselves will bring inflation, even though most economies are working below full capacity. The reasoning uses the tools of the fiscal theory of the price level (FTPL; see Cochrane, 2021). The argument is as follows: Currency is valued by the citizenry because the fiscal authority accepts currency to cancel fiscal obligations. Under this theory, the fiscal authority sets the nominal value of debt and the real value of fiscal surpluses. Thus, the price level accommodates for the intertemporal government budget constraint to hold. Hence, if agents perceive that the government cannot raise enough fiscal surpluses in the future, inflation goes up and the effect of the fiscal stimulus is zero. Two questions come to mind. First, to work, this mechanism needs some form of propagation channel so that demand actually rises more rapidly than supply. The propagation channel used to be wage inflation in the 1970s and 1980s. Currently, we do not see wage inflation, but a disconnection between price and wage dynamics.[1] So, unless wage inflation is triggered, we doubt that inflation (significantly above the current targets) will be a persistent phenomenon in the near future.

We also think that the probability of inflation due to high public debt is not high right now. Let us think again of the FTPL. Let us focus on the euro area, composed by heterogeneous countries. Asymmetry is the key. The price level in the EU depends on the fiscal position of the big countries, which is sound. Thus, according to the FTPL we will not see inflation in the foreseeable future. The problem is debt sustainability in countries like Spain, and for that we need policies to raise productivity and foster growth (see, for instance, de la Fuente, 2021).

Finally, we should point out that the NGEU fund cannot be thought of as a fiscal stimulus in the traditional sense. Its aim is not increasing demand but supply. This is the right policy in the European Union at the moment. As a matter of fact, it is a plan for sectoral transformation in the light of rapid climate change and the fierce competition of the US and China in sectors like semiconductors, 5G, electric cars and the like.

## Fiscal impulse to strengthen the Single Market and coordinate supply-side policies

We are currently in a situation in which a short- and medium-term shock has taken a significant toll on the EU economy. COVID-19 has shown that the incomplete design of European institutions makes a coordinated quick response very difficult. The uneven impact of the coronavirus pandemic and fiscal problems in many countries threatens the Single Market while populist responses to the crisis threaten the European project itself. At the same time, in the long term, we have to face, simultaneously, the digital and ecological transitions. The challenges facing the EU both in the short and long term share the fact that they are market failures. COVID-19 is a negative externality, just like pollution and climate change. In the digital sector, the great revolution is that new technologies have almost negligible marginal costs. The large companies in the sector are gigantic monopolies that actively eliminate competition where it arises, often by the expeditious way of buying emerging rivals. The other major failure – in this case, institutional – faced by all countries is that corporations have great international mobility, so that, in the absence of coordination, the competition to capture tax bases leads to an inefficiently low level of corporate taxation. Moreover, it distorts competition as small and medium-sized companies are less mobile. In addition to the problems described, the EU has other problems derived from its incomplete institutional design, which means that risk sharing is limited. This, in turn, slows down economic integration. Finally, the power of the European Council introduces a non-cooperative bargaining element into the EU's decision-making process, which, as demonstrated by Persson and Tabellini (1996), leads to inefficient allocations.

1 See Bobeica et al. (2019) for the EU case and Stansbury and Summers (2020) for the US.

The NGEU fund is a bold step in two dimensions. First of all, it should imply the creation of a common sectoral policy. Prserving the Single Market requires a coordinated effort in sectoral transformation. The most pressing concerns in this respect are twofold. First, the possible delays in implementing all recovery plans designed by EU members. The second is the typical question that arises when governments implement industrial policies: Are we picking the right winners? For instance, Spain and other countries have high hopes for hydrogen technology. A deep communication between the private economy and governments is needed to really unleash all the potential of that technology given the particularities of each country. This is not to say that one should not pick winners. For instance, China and the US have become leaders in the electric cars or semiconductors sectors because their governments committed to boost them. In our view, the success of the NGEU fund can be expected to strongly depend on the cooperation framework between regional governments and the industry. For instance, the regional Basque government (Spain) has designed its own recovery plan, which appears to be very well connected to industries located in the region.

Second, the NGEU fund is being financed with bonds issued by the Commission, which will give depth to the common currency, something very much needed to foster capital and banking integration within the EU (see Brunnermeier and Reis, 2019). Financing this debt brings the issue of tax policy. It is important that the tax revenues devoted to paying that debt are raised by the Commission. Otherwise, the common debt will be perceived as very risky. The fact that those tax revenues should be raised by the Commission turns our attention to corporate taxation. As argued in Díaz (2020), corporate taxation is a sort of industrial policy. This is why progress should be made towards creating a common corporate taxation as a complement to facilitate the Next Generation Fund to achieve its promised goals.
